# Psychometric properties of the Persian version of the Second Victim Experience and Support Instrument

**DOI:** 10.1002/nop2.1713

**Published:** 2023-04-09

**Authors:** Hamid Sharif‐Nia, Nasrin Hanifi

**Affiliations:** ^1^ Psychiatry and Behavioral Sciences Research Center, Addiction Institute Mazandaran University of Medical Sciences Sari Iran; ^2^ Department of Nursing, Amol Faculty of Nursing and Midwifery Mazandaran University of Medical Sciences Sari Iran; ^3^ Zanjan Nursing and Midwifery School Zanjan University of Medical Sciences (ZUMS) Zanjan Iran

**Keywords:** Iran, medical errors, reliability, second victim, validity

## Abstract

**Aim:**

This study was designed to characterize the psychometric properties of the Persian version of the Second Victim Experience and Support Instrument (P‐SVEST).

**Design:**

This study was a methodological and cross‐sectional study.

**Methods:**

The SVEST was back‐translated into Persian and 10 experts assessed its content validity. Construct validity was determined through exploratory factor analysis (EFA) and confirmatory factor analysis (CFA) with a total of 754 critical care and emergency nurses.

**Results:**

The results of exploratory factor analysis showed that the P‐SVEST had four factors. These four factors accounted for 51.67% of the total variance. Also, these factors were confirmed by confirmatory factor analysis (root mean square error of approximation = (90%. confidence interval) = 0.058 [0.045, 0.071], goodness‐of‐fit index = 0.932, comparative fit index = 0.956, non‐normal fit index = 0.918, incremental fit index = 0.957 and Tucker–Lewis index = 0.944). Coefficients of Cronbach's alpha, McDonald's omega, composite reliability and maximum reliability for all of the factors were >0.7, demonstrating satisfied internal consistency.

## 1 INTRODUCTION

Health care professionals face stressful situations and a high workload all the time. These working conditions can be frustrating for staff and increase medical errors (Griffiths et al., 2020; Sturm et al., 2019). Medical errors and adverse events are inevitable in any health care organization. These issues often have catastrophic effects on the patient, health care providers and the organization. Although most health care professionals experience medical errors (Khammarnia et al., 2021; Mosadeghrad et al., 2020), nurses are more prone to medical errors due to their high workload (Di Muzio et al., 2019).

Outcomes of medical errors and the patient have adverse effects on health care professionals (Cabilan & Kynoch, 2017; Finney et al., 2021). Following a medical error, the patients and their families are considered the first victims, and the health care professionals who are injured after the incident are the second victims (Busch et al., 2020). The feeling of health and well‐being is affected in the second victim after the safety events. The victims experience psychological and physical distress and endure a high level of stress (Chan et al., 2017; Ozeke et al., 2019; Miller et al., 2019; Van Gerven, Deweer, et al., 2016; Van Gerven, Vander Elst, et al., 2016). After safety incidents, adverse outcomes in health care professionals include physiological disorders, sleep disorders, occupational dysfunction, burnout, powerlessness, decreased job satisfaction, decreased job confidence, guilt, anger and shame, anxiety about punishment, job loss and litigation (Bari et al., 2016; Garrouste‐Orgeas et al., 2015; Joesten et al., 2015; Lee et al., 2019; Miller et al., 2019; Mohsenpour et al., 2017; Tawfik et al., 2018; Van Gerven, Deweer, et al., 2016; Van Gerven, Vander Elst, et al., 2016). Health care professionals who feel like a second victims need support (Schrøder et al., 2019). However, organizations' support resources are often insufficient to prevent and reduce work‐related injuries. Lack of organizational support leads to reduced patient safety and consequently, patient injury and injury to health care providers (Farokhzadian et al., 2018; Rinaldi et al., 2016). The second victim's mental, physical and occupational distress reflects a culture of punitive safety and a lack of organizational support (Burlison et al., 2017; Quillivan et al., 2016).

It is essential to examine the experiences of people involved in safety incidents with a valid and reliable instrument. Using a valid and reliable instrument can help implement programmes to increase support for second victims and increase patient safety. The Second Victim Experience and Support Instrument (SVEST) was originally developed by Burlison et al. (2017). This instrument measures health care professionals' distress after an error occurs and assesses the quality of support resources and the outcomes for staff. The instrument has been validated in different countries (Brunelli et al., 2021; Chen et al., 2019; Kim et al., 2020; Knudsen et al., 2021; Koca et al., 2022; Mohd Kamaruzaman et al., 2022; Santana‐Domínguez et al., 2021, 2022; Scarpis et al., 2021). Ajoudani et al. also evaluated the psychometric properties of this instrument in Urmia, Iran, on 298 nurses working in general wards. In the study of Ajoudani et al. (2021), exploratory factor analysis (EFA) was not performed and only confirmatory factor analysis (CFA) was performed. Nurses in intensive care units (ICUs) and emergency departments (EDs) make more errors than nurses in general wards, so they experience more of the second victim experience. On the other hand, it seems necessary to perform EFA to find latent variables in this instrument based on Iranian culture. Therefore, this study was designed to assess the psychometric properties of the Persian version of the Second Victim Experience and Support Instrument (P‐SVEST).

## 2 METHODS

### 2.1 Research design

This methodological cross‐sectional study was conducted on the critical care and emergency nurses from September to October 2021 to evaluate validity and reliability of the P‐SVEST. The inclusion criteria of the participants in this study were willingness to participate in the study, employment in critical care units and emergency departments and having 2 years of work experience and medical error experience.

### 2.2 Measures

There were two sections in the instrument survey. The first section consisted of items regarding participants' profiles, such as age, gender and workplace unit. In the second section, the 29‐item SVEST was used to measure the feeling of the second victim and the support received by the participants (Burlison et al., 2017). Items were measured on five‐point Likert scales, with scores ranging from 1 “strongly disagree”to 5 “strongly agree”. Also, seven support options were included in this instrument, and the desirability of support options was measured by items anchored on a five‐point Likert scale ranging from 1 “strongly do not desire”to 5 “strongly desire”.

### 2.3 Procedure

Regarding the validity of translation process, the SVEST was translated based on the standards recommended in the guidelines (Beaton et al., 2002). Initially, written permission for the SVEST was obtained from the developer of the scale, Dr. James M. Hoffman, via email. Subsequently, based on the forward–backward translation technique, two English–Persian translators were asked to translate the SVEST into Persian. The two translators translated the SVEST into the Persian version independently. Afterwards, these two Persian versions of the SVEST were reviewed and commented on by a group of experts, including some authors of this article and another two professional translators, to form a single P‐SVEST. Finally, the single P‐SVEST was back‐translated to English by a Persian–English translator, and the translation accuracy was confirmed by a group of experts.

### 2.4 Content validity

The face validity was obtained by providing the instrument to 20 nurses and asking them to identify the sentences and phrases that are vague. Content validity was performed by 10 experts (six faculty members from the department of intensive care nursing and emergency nursing and four nursing managers) who used the four‐point method from 1 being irrelevant to 4 being highly relevant to score each item of the P‐SVEST. These experts evaluated the content of the P‐SVEST. Afterwards, the item‐ and scale‐level content validity indexes (I‐CVI and S‐CVI, respectively) were calculated for the P‐SVEST instrument. The acceptable ranges for I‐CVI and S‐CVI are >0.80 and >0.92, respectively (Polit & Yang, 2016). Content validity index correction of random chance agreement was made (Pa) using the formula = [N!/(A! (A! (N A)!)]*0.5 N, ˆ where *N* = no Expert and A = *n* of agreement with good relevance and the statistical calculation of modified Kappa (K* = (CVI‐i‐Pa)/(1‐Pa)) for each item of the tool. The evaluation criteria of the K* were poor: K* values <0.39; moderate: K* values = 0.40–0.59; good: K* values = 0.60–0.74; and excellent: K* values > 0.74; Vahid et al., 2015; Table 1).

**TABLE 1:** Content validity analysis by item.

Item	Number of agreement	I‐CVI	Pc	K*	Evaluation
1.	10	1	0.001	1	VALID
2.	10	1	0.001	1	VALID
3.	10	1	0.001	1	VALID
4.	10	1	0.001	1	VALID
5.	8	0.8	0.044	0.79	VALID
6.	10	1	0.001	1	VALID
7.	8	0.8	0.044	0.79	VALID
8.	10	1	0.001	1	VALID
9.	8	0.8	0.044	0.79	VALID
10.	8	0.8	0.044	0.79	VALID
11.	8	0.8	0.044	0.79	VALID
12.	8	0.8	0.044	0.79	VALID
13.	10	1	0.001	1	VALID
14.	10	1	0.001	1	VALID
15.	8	0.8	0.044	0.79	VALID
16.	10	1	0.001	1	VALID
17.	10	1	0.001	1	VALID
18.	10	1	0.001	1	VALID
19.	8	0.8	0.044	0.79	VALID
20.	10	1	0.001	1	VALID
21.	10	1	0.001	1	VALID
22.	10	1	0.001	1	VALID
23.	10	1	0.001	1	VALID
24.	10	1	0.001	1	VALID
25.	8	0.8	0.044	0.79	VALID
26.	8	0.8	0.044	0.79	VALID
27.	8	0.8	0.044	0.79	VALID
28.	10	1	0.001	1	VALID
29.	10	1	0.001	1	VALID

Abbreviations: CVI‐I, Content validity per each item; K*, modified Kappa; Pa, random‐likelihood agreement.

### 2.5 Construct validity and reliability

This study conducted both EFA and CFA to confirm the factorial structure and the construct validity and reliability of the P‐SVEST. The sample size should be at least 200 cases for factor analysis (Beaton et al., 2002). A total of 754 nurses were recruited via online data gathering. Online data gathering was performed for this section where the online questionnaire was created via Porsline and its URL link was sent by social networking applications such as Telegram channel or WhatsApp groups. Data were then extracted into an Excel file from the Porsline.

For data analysis, the dataset (*n* = 754) was randomly divided into two parts. The data were split randomly using: Data ‐ > Select Cases and “Random sample of cases” was chosen. The first dataset (*n* = 377) was used to conduct EFA using SPSS version 27, and the second one (*n* = 377) was used to conduct CFA using AMOS version 27. This study employed maximum‐likelihood EFA with Promax rotation, the Kaiser–Meyer–Olkin (KMO) > 0.8 and Bartlett's test of sphericity (*p* < 0.05) to assess the relevance and appropriateness of the data for conducting the factor analysis. The EFA was run by Pearson matrices.

The factorial structure of P‐SVEST was based on the criteria of eigenvalues >1, communalities >0.2 and scree plots. Moreover, the factor loading for each item in the extracted factors should be >0.3 (Çokluk & Koçak, 2016). Next, we conducted maximum‐likelihood CFA to validate the factorial structure extracted from EFA. The model fit was assessed through a number of fit indices, including Chi‐square (*χ* ^2^) test, Chi‐square (*χ* ^2^)/degree of freedom (df) ratio <4, goodness‐of‐fit index (GFI) > 0.9, comparative fit index (CFI) > 0.9, normed fit index (NFI) > 0.9, relative Fit Index (RFI) > 0.9, incremental fit index (IFI) > 0.9, Tucker–Lewis's index (TLI) > 0.9, standardized root mean square residual (SRMR) < 0.09 and root mean square error of approximation (RMSEA) < 0.08. The P‐SVEST was evaluated through its convergent validity and discriminant validity.

The convergent and divergent validity of the P‐SVEST were estimated using Fornell and Larcker's approach (Fornell & Larcker, 1981). For convergent validity, composite reliability (CR) should be higher than 0.7, and average variance extracted (AVE) should be >0.5 (Sharif Nia et al., 2015). This study also assessed the construct reliability over its internal consistency (Cronbach's alpha and McDonald's omega; Javali et al., 2011), composite reliability (CR) and maximum reliability (MaxR). To achieve acceptable construct reliability, Cronbach's alpha and McDonald's omega, CR and MaxR should be >0.7 (Sharif Nia et al., 2021).

### 2.6 Multivariate normality and outliers

Both univariate and multivariate normality of the data was evaluated in this study. The univariate distributions were tested for outliers, skewness and kurtosis. Also, the multivariate normality was assessed using Mardia's coefficient of multivariate kurtosis and Mardia's coefficient. Mardia's coefficient of multivariate kurtosis <8 can be considered indicative of departure from multivariate normality (Henseler & Fassott, 2010). Moreover, the outliers of the multivariate were detected using Mahalanobis distance (*p* < 0.001; Leys et al., 2018).

### 2.7 Ethical considerations

This study was conducted after obtaining the code of the research ethics committee (IR.ZUMS.REC.1399.335) from Zanjan University of Medical Sciences. On the first page of the electronic instrument, the study's objectives were explained to the participants. If the participants were willing to participate in the survey, they would sign the electronic consent form and complete the instrument.

## 3 RESULTS

### 3.1 Participants' profiles

In total, 754 Iranian nurses participated in this study, including 203 males and 552 females. Most of the participants were in the age group of 31–40 years with 6–10 years of work experience (Table 2).

**TABLE 2:** Respondent characteristics (*N* = 754).

Characteristics	Categories	*n*	%
Gender	Male	203	26.92
	Female	551	73.08
Marital status	Single	197	26.12
	Married	557	73.87
Education	Bachelor	705	93.5
	Master science	49	6.5
Department	ICU	247	32.76
	CCU	108	14.32
	Haemodialysis	44	5.83
	PICU	31	4.11
	NICU	89	11.8
	ED	235	31.17
Employment status	Casual employees	376	49.87
	Fixed employment contracts	211	27.98
	Permanent full‐time employment	167	22.15
Age	22–30	262	34.75
	31–40	310	41.11
	41–50	182	24.13
Work experiences (years)	1–5	196	25.99
	6–10	262	34.75
	11–15	94	12.47
	16–20	118	15.6 5
	21–26	84	11.14

Abbreviations: CCU, Coronary Care Unit; ED, Emergency Department; ICU, Intensive Care Unit; NICU, Neonatal Intensive Care Unit; PICU, Paediatric Intensive Care Unit.

### 3.2 Validity and reliability

Table 3 shows the results of the EFA with Promax rotation (*n* = 377) on the P‐SVEST. The results showed that the KMO was 0.830, and Bartlett's test of sphericity was statistically significant (*p* < 0.001, 1955.755, df = 105), indicating the relevance and appropriateness of the data for conducting the factor analysis. Four factors were extracted that consisted of 15 items and explained 51.67% of the total variance. Also, 14 items were removed due to the communalities of <0.2 and factor loading of <0.3.

**TABLE 3:** The result of EFA and internal consistency on the four factors of the P‐SVEST (*N* = 377).

Factor	Items	Mean (SD)	Factor loading	*h* ^2^	λ	% variance	Internal consistency
Physical distress	Q_6_. My experience with these occurrences can make it hard to sleep regularly	3.09 (1.12)	0.897	0.757	2.394	15.96	α = 0.869 Ω = 0.875 AIC = 0.622
	Q_7_. The stress from these situations has made me feel queasy or nauseous.	3.16 (1.08)	0.829	0.699			
	Q_8_. Thinking about these situations can make it difficult to have an appetite.	3.02 (1.07)	0.791	0.599			
	Q_5_. The mental weight of my experience is exhausting.	3.45 (1.00)	0.526	0.506			
Psychological distress	Q_1_. I have experienced embarrassment from these instances.	3.55 (1.02)	0.896	0.655	2.070	13.80	α = 0.792 Ω = 0.792 AIC = 0.488
	Q_2_. My involvement in these types of instances has made me fearful of future occurrences.	3.56 (1.01)	0.766	0.580			
	Q_4_. I feel deep remorse for my past involvements in these events.	3.12 (1.05)	0.669	0.617			
	Q_3_. My experiences have made me feel miserable.	2.85 (1.05)	0.483	0.523			
Hospital support	Q_17_. My organization understands that those involved may need help to process and resolve any effects they may have on care providers.	3.06 (1.14)	0.798	0.632	1.855	12.37	α = 0.702 Ω = 0.705 AIC = 0.351
	Q_18_. My organization offers a variety of resources to help me get over the effects of involvement with these instances.	3.22 (1.17)	0.717	0.539			
	Q_13_. I feel that my supervisor treats me appropriately after these occasions.	3.10 (0.99)	0.610	0.381			
	Q_16_. I feel that my supervisor evaluates these situations in a manner that considers the complexity of patient care practices.	3.00 (0.97)	0.577	0.325			
Professional self‐efficacy	Q_24_. After my experience, I became afraid to attempt difficult or high‐risk procedures.	3.24 (0.99)	0.933	0.791	1.432	9.54	α = 0.712 Ω = 0.715 AIC = 0.346
	Q_23_. My experience makes me wonder if I am not really a good health care provider.	2.83 (1.04)	0.605	0.444			
	Q_26_. My experience with these events has led to a desire to take a position outside of patient care.	2.97 (1.12)	0.443	0.238			

Abbreviations: AIC, Average inter‐item correlation; *h* ^2^, Communalities; SD, Standard deviation; α, Alpha; λ, Eigenvalues; Ω, Omega.

**FIGURE 1:**
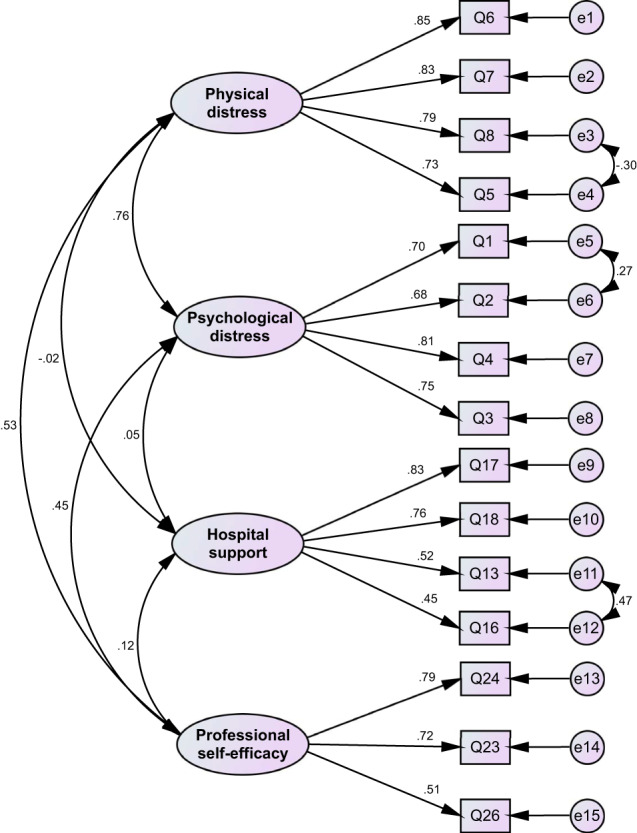
Factor structure of the P‐SVEST with correlations among the five factors, standardized factor loadings, and error terms.

Next, maximum‐likelihood CFA (*n* = 377) was conducted to validate the factorial structure obtained from EFA. As shown in Figure 1, to improve the model, three pairs of measurement error were allowed to co‐vary freely (i.e. e_3_ to e_4_, e_5_ to e_6_ and e_11_ to e_12_). The factor loadings for all items ranged from 0.45 to 0.85 and were statistically significant. The final four‐factor model fits the data well after reviewing the modification indices (*χ* ^2^(81) = 1630.195, *p* < 0.001, *χ* ^2^/df = 2.015, GFI = 0.932, CFI = 0.956, NFI = 0.918, IFI = 0.957, TLI = 0.944 and RMSEA (90% CI) = 0.058 [0.045, 0.071]).

Coefficients of Cronbach's alpha, McDonald's omega, CR and MaxR for all of the factors were >0.7, indicating the satisfied internal consistency and construct reliability. Moreover, the AVE for three factors was less than the required threshold of 0.5 or MSV, and AVE is a strict measurement for convergent validity. CR more than 0.7 can be used to assess convergent validity in psychological studies. Therefore, the CR convergent validity achieved in this study was >0.7 for both factors (Table 4).

**TABLE 4:** The indices of the convergent, discriminant validity in the CFA model (*n* = 377).

	CR	AVE	MSV	MaxR (H)
Physical distress	0.877	0.640	0.571	0.883
Psychological distress	0.824	0.540	0.571	0.832
Hospital support	0.746	0.438	0.013	0.811
Professional self‐efficacy	0.718	0.467	0.276	0.754

## 4 DISCUSSION

This study aimed to determine the psychometric properties of the P‐SVEST instrument. Using EFA, four factors were extracted in the P‐SVEST instrument, which explained 51.67% of the total variance. Based on the Iranian culture, P‐SVEST identified four factors: physical distress, psychological distress and institute support with four items each and professional self‐efficacy factor with three items. Eventually, the CFA results showed that the psychometric properties of P‐SVEST had good validity and reliability.

In the original version of the SVEST, seven factors and two outcomes were confirmed by CFA. Based on the EFA results, 14 items from the initial version of SVEST were removed due to the communalities <0.2 and factor loading <0.3. In this study, the two factors of physical distress and psychological distress, according to the evidence, are the physical and psychological symptoms that the second victim experiences due to high stress (Bari et al., 2016; Chan et al., 2017; Garrouste‐Orgeas et al., 2015; Joesten et al., 2015; Lee et al., 2019; Miller et al., 2019; Mohsenpour et al., 2017; Ozeke et al., 2019; Tawfik et al., 2018; Van Gerven, Deweer, et al., 2016; Van Gerven, Vander Elst, et al., 2016). The items of these two factors are consistent with the original version of the instrument. All four items of the original version of colleague support were removed. In the Supervisor Support Factor of the original version, the items “My supervisor's responses are fair” and “My supervisor blames individuals” of the original version were removed. In the institution support factor of the original version, the item “The concept of concern for the well‐being of those involved in these situations is not strong at my organization” was removed from the original version. In the non‐work‐related support dimension, both items of the original version were removed. Also, in the professional self‐efficacy factor of the original version, the items “Following my involvement, I experienced feelings of inadequacy regarding my patient care abilities” and “These situations do not question my professional abilities” were removed. In the turnover intentions dimension of the original version, the item “Sometimes the stress from being involved with these situations makes me want to quit my job” was removed. In addition, in the absenteeism dimension, both items of the original version were removed. In the present study, items 13 and 16 of the institution support factor and items 17 and 18 of the institution support factors were included in one factor. Accordingly, in this study, this factor was called hospital support. The support of the organization to the staff after an error occurs leads to a reduction in the occurrence of repeated errors and an increase in patient safety. In the present study, this factor was called hospital support with three items (Farokhzadian et al., 2018; Rinaldi et al., 2016; Schrøder et al., 2019).

The feeling of professional inefficiency after an error is associated with feelings such as occupational dysfunction, burnout, powerlessness, decreased job satisfaction and decreased job confidence (Bari et al., 2016; Garrouste‐Orgeas et al., 2015; Joesten et al., 2015; Lee et al., 2019; Miller et al., 2019; Mohsenpour et al., 2017; Tawfik et al., 2018; Van Gerven, Deweer, et al., 2016; Van Gerven, Vander Elst, et al., 2016). In this study, the professional self‐efficacy factor was evaluated with three items.

In the study of Chen et al. (2019), eight items were removed, and six factors were extracted in the exploratory analysis for the Chinses version of SVEST. In the original version of the SVEST, seven factors and two outcomes were confirmed by CFA. Ajoudani et al.'s study, which was conducted on 298 nurses in Iran, confirmed seven factors and two outcomes by conducting CFA. The participants in the study of these researchers were nurses working in general wards, also, EFA was not conducted to find latent variables (Ajoudani et al., 2021). In Tan et al.'s study, 21 items in four factors were extracted using the maximum‐likelihood and Promax rotation methods (Tan et al., 2020). In the study of Strametz et al. (2021), 11 factors were extracted using the principal component analysis. Santana‐Domínguez et al.'s study five factors were extracted using the parallel analysis of the EFA (Santana‐Domínguez et al., 2022). In some psychometric studies of this instrument, EFA was not performed and only CFA was applied. Also, some studies have tested the dimensions of the original version (Ajoudani et al., 2021; Knudsen et al., 2021; Pieretti et al., 2022). In our study, unlike the original version, physical distress was included in factor 1 and psychotic distress was included in factor 2. In addition, unlike the original version, in the study of Chen et al. (2019), factor 1 was allocated to institutional support and supervisor support, and factor 2 was allocated to self‐efficacy and negative work outcomes occupational. This  change in factors and items in studies reflects the cultural differences of different countries in the experience of feeling the second victim of staff.

According to this study results, most nurses who experience a patient's safety event in Iran reported physical and psychological distress. Consistent with the results of our study, in other studies, people experience these symptoms following a medical error (Zhang et al., 2019; Zheng et al., 2022). According to Iranian nurses, support resources after patient safety events are mostly related to the supervisor and the institution. Unlike previous studies, colleague support and non‐work‐related support have a negligible effect on improving the condition of feeling like a second victim (Chen et al., 2019; Quillivan et al., 2016; Santana‐Domínguez et al., 2021). In Iranian culture, due to the lack of nurses, absenteeism is almost impossible. Therefore, contrary to previous studies, this factor was removed in our study (Brunelli et al., 2021; Burlison et al., 2017; Kim et al., 2020; Pieretti et al., 2022). Since having a job is a basic need for Iranian citizens, nurses have to be present at work after these events, even in the worst physical, mental and professional self‐efficacy conditions. Therefore, in these circumstances, the support of the supervisor and the institute creates more peace for the nurses than the support of colleagues and family members. Otherwise, they cannot ensure their job security.

In the present study, all factors with Cronbach's alpha >0.7 had good reliability. However, studying the psychometric of the Danish version of SVEST, Knudsen et al. (2021) showed that Cronbach's alpha did not have adequate reliability in the factors of colleague support (0.4) and institute support (0.68). Elsewhere, studying the original version (Burlison et al., 2017), the Korean SVEST version (Kim et al., 2020) and the Argentinian SVEST study (Brunelli et al., 2021) reported poor‐to‐questionable Cronbach's alpha coefficients for these two factors. In the present study, these two factors were used to remove the EFA phase.

The results of this study demonstrated that nurses in intensive care units and the emergency department endure psychological and physical distress after patient safety events, which is consistent with other studies (Quillivan et al., 2016; Zhang et al., 2019). However, the consequences of the second victim experience for Iranian nurses are different in some countries (Brunelli et al., 2021; Kim et al., 2020). Also, support sources are different from the perspective of Iranian nurses (Brunelli et al., 2021; Burlison et al., 2017; Kim et al., 2020).

In this study, due to the removal of three factors and 14 items from the original instrument, the generalizability of the instrument is limited. On the other hand, because the data collection method was online, nurses from most provinces of Iran participated in this study. Therefore, this instrument can be used to assess the experience of the second victim and nurses' support resources in critical care units and emergency departments of Iran. In addition, since the participants in this study only include the nursing community, the generalizability of the P‐SVEST to other professional health care providers is limited.

## 5 CONCLUSION

The P‐SVEST instrument examines nurses' second victim experience and support resources with four factors. According to the cultural and environmental conditions of Iranian hospitals, the second victim experience and support resource can be measured with the help of the 15 items of approximately 50%, but it needs to be localized in other studies with more items and appropriate to the environment and culture of the Iranian society. Evaluating the psychometric properties of this instrument on 754 nurses showed that the instrument has construct validity, content validity and internal consistency. For future studies, it is recommended that managers provide the necessary support resources for the second victims by examining the extent of their distress because nurses' physical and mental health plays an essential role in ensuring patients' safety.

## AUTHOR CONTRIBUTIONS

Study design: Nasrin Hanifi and Hamid Sharif Nia; data gathering: Nasrin Hanifi; statistical analysis and interpretation of the data; Nasrin Hanifi; drafting of the manuscript: Nasrin Hanifi; critical revision of the manuscript: Nasrin Hanifi and Hamid Sharif Nia.

## CONFLICT OF INTEREST STATEMENT

The authors declare no conflict of interest.

## ETHICAL APPROVAL

This study was conducted after obtaining the code of the research ethics committee (IR.ZUMS.REC.1399.335) from the Zanjan University of Medical Sciences. The study was carried out in accordance with the 1964 Helsinki Declaration and the ethical standards of the National Research Committee.

## Data Availability

The data that support the findings of this study are available on request from the corresponding author.
